# Hybrid perovskite solar cells fabricated from guanidine hydroiodide and tin iodide

**DOI:** 10.1038/s41598-017-05317-w

**Published:** 2017-07-10

**Authors:** Hironobu Ishibashi, Mikimasa Katayama, Senku Tanaka, Toshihiko Kaji

**Affiliations:** 1 Department of Applied Physics, Tokyo University of Agriculture and Technology, Tokyo, 184-8588 Japan; 2 Faculty of Science and Engineering, Kindai University, Osaka, 577-8502 Japan

## Abstract

For the search of new metal-halide perovskite solar cell materials, tolerance factors are calculated from the ionic radius of each site and are often utilized as the critical factors to expect the materials forming perovskite structure. As one of such amine hydrohalides, guanidine hydroiodide (GI) is reported not to react with PbI_2_. However, in this paper, we report the product of GI and SnI_2_ reaction, its visible light absorption, X-ray diffraction, and its solar cell operation, in spite of the more disadvantageous tolerance factor of SnI_2_ than PbI_2_. We also report the thermal stability of GI, enabling precise control of vacuum deposition, and utilization of co-evaporant induced crystallization method during the vacuum evaporation of the SnI_2_ film, which resulted in enlarging the SnI_2_ crystals and improving the short circuit current density of the solar cell.

## Introduction

Recently, perovskite solar cells attract much attention because of their high efficiency and ease of fabrication. In perovskite solar cells, organic halogenated perovskites are used as energy conversion materials, their compositions are generally represented as ABX_3_: A site is an organic amine, B site is a metal, and X site is a halogen. To make this structure stable, the size of each ion is important and the stability can be estimated from tolerance factor. For the search of new perovskite materials, tolerance factor has been calculated in various combinations^1–4^ of organic materials and metals. From this calculation, not only the standard organic amines of methylamine (MA) and formamidine (FA), but also various organic amines are reported^3, 5^ on their possibilities to form perovskite structure and to be used as solar cell materials.

One of such prospected organic amines, guanidine (G), shows tolerance factor of almost 1 by combination with PbI_2_ or SnI_2_, similar to the case of ethylamine (EA) because of their larger ionic radii than of MA and FA. For example, these values have been reported^1, 3^ as the followings, tolerance factors of GSnI_3_: 1.051 and GPbI_3_: 1.039 by using each ionic radius of Sn: 115 pm, Pb: 119 pm, guanidine: 278 pm, I: 220 pm. Although perovskite solar cells using EAI and PbI_2_ have been already reported^4^, in contrast, Yang *et al*. reported that GI and PbI_2_ did not form perovskite; whereas the open circuit voltage of mainly MAPbI_3_ composed cell was improved by adding GI^6^. Predicting by the tolerance factor, GI and SnI_2_ cannot form perovskite as well as the case of PbI_2_. In this paper, however, we will report that GI reacted well with SnI_2_ and the product absorbed wider visible light region than the reactants did. We succeeded in fabricating and operating the solar cell using only guanidine for the A site and Sn for the B site.

## Results and Discussion

**Figure 1:**
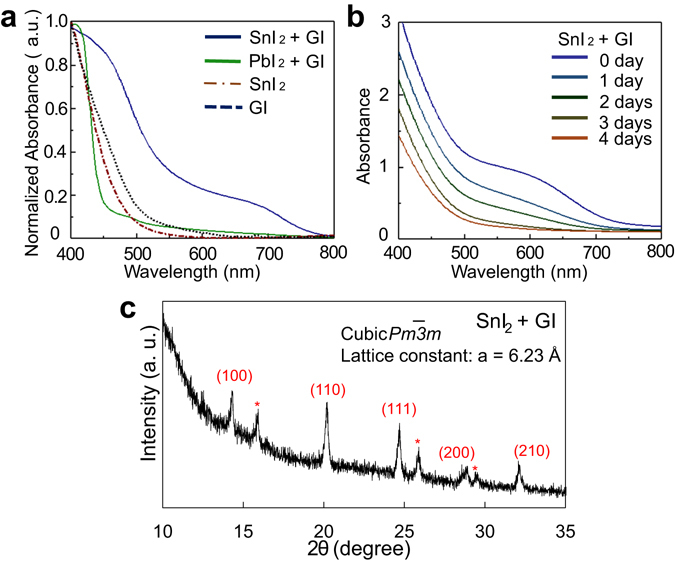
Characteristics of films fabricated from solution. (**a**) Absorbance spectra of PbI_2_ + GI, SnI_2_ + GI, SnI_2_, and GI films formed from acetone solutions. (**b**) Degradation of absorbance spectra of SnI_2_ + GI film. (**c**) X-ray diffraction pattern of SnI_2_ + GI film.

First, the reactivity of GI and inorganic material (SnI_2_ or PbI_2_) was examined by drop-coating. Films were formed from acetone solution on quartz substrates and absorbance spectra of the films were measured by an ultraviolet and visible spectrophotometer. As shown in Fig. 1a, an absorption edge around 800 nm and a shoulder around 700 nm were observed for the product of SnI_2_ and GI. In contrast to this clear change, no increase of visible light absorption by the reaction was observed for PbI_2_ and GI as same as reported^6^. In Fig. 1b, air stability of the product was evaluated. Although the film was preserved in the dry air, its visible absorption decreased day by day, probably because of Sn oxidization.

To distinguish whether this product is perovskite or not, we performed X-ray diffraction (XRD) measurement. As shown in Fig. 1c, the result showed clear peaks that can be attributed to *Pm-3m* space group of cubic perovskites and confirms that the formation of 3d -perovskite of GSnI_3_. Smaller peaks marked * may indicate superlattice, which imply the partial degradation of the cubic perovskite structure because of large tolerance factor or air degradation. A rough calculation using each ionic radius can reasonably explain the lattice constant of 6.23 Å. When simply supposing the sequence of AXA face-centred lattice, ABA body-centred lattice, and BXB side of the simple cubic lattice instead of ABX_3_, lattice constant can be calculated as 7.04, 4.54, and 6.70 Å, respectively. The average of 6.09 Å is appropriately near the experimental value of 6.23 Å, although further study is necessary to clarify more accurate crystal structure of GSnI_3_.

Next, we fabricated solar cell devices. We attempted fabricating the devices by vacuum deposition method. We found that GI can be normally deposited by vapor evaporation, different from the depositions of hydrohalides of MA and FA, which often has problems by pyrolysis and diffusion of the materials, such as deterioration of vacuum, serious contamination to the vacuum equipment^7^, and evolution of toxic hydrogen cyanide dictated on FAI^8^. In our vacuum system, MAI and FAI also showed contaminations to the wide area of the system. However, when we tried depositing GI, on the other hand, its vacuum deposition was stably controlled under high vacuum of 1–3 × 10^−3^ Pa without such problems. We consider that the reason of this higher thermal stability of GI than MAI and FAI is because methylamine without iodine ion is a gas substance at room temperature and that formamidine itself cannot be isolated from the halide, whereas guanidine is solid.

**Figure 2:**
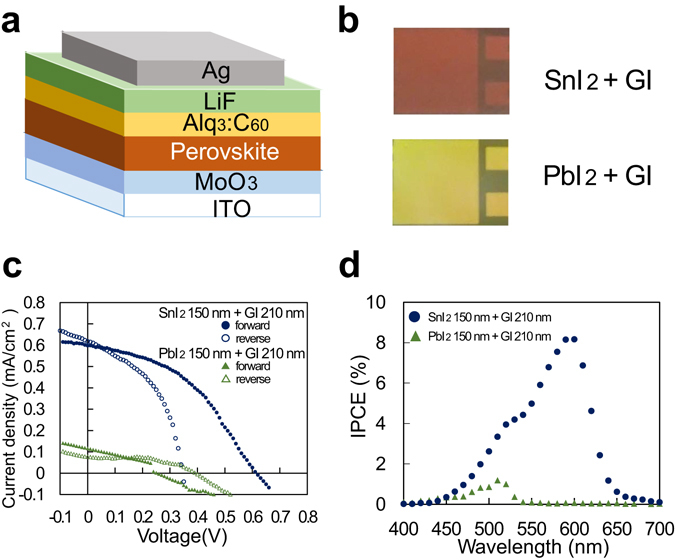
Solar cells fabricated by two-step method of vacuum evaporation using SnI_2_ or PbI_2_ as a metal halide and GI as an amine halide. (**a**) Device structure, (**b**) picture of the films, (**c**) *J*-*V* curve, and (**d**) IPCE.

A highly efficient Sn-based perovskite device of the reverse structure type without the charge transport layer of TiO_2_ porous film has been reported^9–11^. Thus, we fabricated the device of the reverse structure type as depicted in Fig. 2a. The electron transport layer and hole transport layer were chosen referring to the materials used for vacuum deposition of organic solar cells^12–15^ as shown in Fig. 2a. All layers were successively formed by vacuum deposition on pre-patterned indium tin oxide coated glass to fabricate the solar cells. Perovskite layer was formed by two-step method: SnI_2_ or PbI_2_ layer deposition was followed by GI deposition. Throughout these perovskite layer depositions, the substrate temperature was kept at 70 °C. During all the vacuum evaporation, the degree of vacuum was 1–3 × 10^−3^ Pa, and the deposition rate was 0.5 Å/s for GI and 1.0 Å/s for other materials. Solar cell performances of the devices fabricated were measured in N_2_ filled glove box without taken out to the atmosphere.

After the optimization of the perovskite layer, we reached the ratio of GI: 210 nm and SnI_2_: 150 nm in nominal thickness. Figure 2b,c and d show the photographs of the fabricated devices, the *J*-*V* curve of the device with 1 cm^2^ area measured under the AM1.5 1sun simulated illumination, and incident photon to current conversion efficiency (IPCE) of the same device but with 0.04 mm^2^, respectively. In Fig. 2b, the color of SnI_2_ + GI is brown unlike the orange color of SnI_2_. In Fig. 2c forward and reverse represent the direction of voltage sweep from −2 V to 2 V and from 2 V to −2 V, respectively. The device parameters of SnI_2_ + GI from the forward sweep were the followings: 0.60 mA/cm^2^ of short-circuit current density (*J*_sc_), 0.62 V of opening circuit voltage (*V*_oc_), 0.42 of fill factor (FF), and 0.16% of power conversion efficiency (PCE). On the other hand, the color of PbI_2_ + GI showed yellow as same as PbI_2_. The operation of PbI_2_ + GI device was unstable, and its performance was much limited to 0.12 mA/cm^2^ of *J*_sc_, 0.26 V of *V*_oc_, 0.36 of FF, and 0.01% of PCE. Judging from the IPCE spectra in Fig. 2d, in which the SnI_2_ + GI device has photo-response up to 650 nm of the long wavelength side, light absorption of the product of SnI_2_ + GI reaction certainly contributed to the current generation of the solar cell. On the other hand, PbI_2_ device has only 450–550 nm of much smaller photo-response, which might be from Alq_3_ or C_60_ in the buffer layer.

Further enhancement of the performance can be usually possible by improving device structure or post-annealing^16, 17^. Especially, crystallinity and particle size of the active layer have great importance on the performance of perovskite solar cells^18–26^. Here, we tried introducing co-evaporant molecules during the vacuum evaporation to induce crystallization of the film in this study. This unique method is originally an improved method of the vacuum deposition for organic films and enables crystallinity of organic films by co-evaporating liquid molecules during vacuum evaporation of the film materials^27, 28^. The liquid promotes surface diffusion of film material molecules, but the liquid itself does not remain on the device substrate because of substrate heating. In the case of organic photovoltaic cells, the cells whose active layers were crystallized by this method indicated improvements of their short circuit current density. Thus, we examined the utility of this unique method for perovskite solar cells, although the film materials in this study are inorganic films of SnI_2_ and PbI_2_, different from the organic materials in past studies.

**Figure 3:**
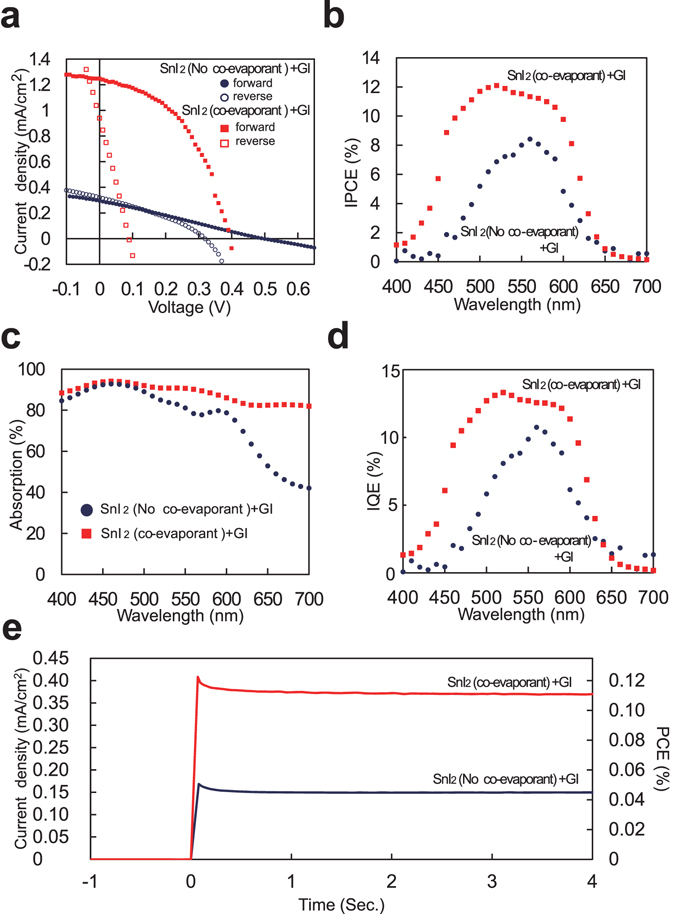
Performance improvements of SnI_2_ + GI solar cells by co-evaporant induced crystallization. (**a**) *J*-*V* curve of 1 cm^2^ cell, (**b**) IPCE, (**c**) absorption spectra, (**d**) IQE, and (**e**) stabilized power output under bias application of 0.30 V.

We used polydimethylsiloxane (PDMS) as the co-evaporant liquid and its evaporation rate was nominally 0.2 A/s. We introduced this liquid only during the deposition of the SnI_2_ film. Other fabrication conditions are almost same as the above; the exception is the thickness of perovskite layer, which was thinned from the device in Fig. 2c, SnI_2_: 150 nm and GI: 210 nm, to the followings, SnI_2_: 100 nm and GI: 140 nm, because the short of the electrodes occurred with thicker devices due to the extremely rough surface formed by the co-evaporant method as shown in later. It implies that further optimization of both active layer and the buffer layer is necessary for the stable production of this type cells. As the result of the introduction of the co-evaporant liquid during the deposition of the SnI_2_ film, short circuit current density improved. As shown in Fig. 3a, *J*_sc_ was 1.25 mA/cm^2^, *V*_oc_, FF, and PCE were 0.40 V, 0.46, and 0.23%, respectively from the forward sweep. Particularly, *J*_sc_ is 4.3 times higher than the device without co-evaporant in Fig. 3a and 2.1 times higher than the device before thinning the film thickness in Fig. 2c. Although large hysteresis was observed, its effect on *J*_sc_ was small enough to compare the difference of the devices. To confirm this performance difference and the operation of GSnI_3_ cells under steady-state, we added stabilized power output measurement^29^ under the bias application of 0.30 V as shown in Fig. 3e. *J*_sc_ and PCE for the device with co-evaporant, 0.37 mA/cm^2^ and 0.11%, were certainly higher than 0.15 mA/cm^2^ and 0.045% for the device without co-evaporant, although the devices in Fig. 3e are the different batch but the same composition from them in Fig. 3a,b,c and d.

**Figure 4:**
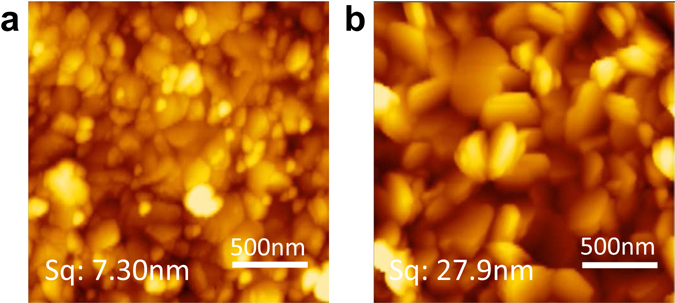
AFM images of PbI_2_ films vacuum deposited (**a**) without co-evaporant, and (**b**) with co-evaporant. Sq is the root mean square height, which is standardized by ISO25178 as one of the parameters representing surface roughness.

The morphological change of the SnI_2_ film by co-evaporant was difficult to measure because the film was easily oxidized and deteriorated in the atmosphere. Therefore, to infer the effect of co-evaporant on SnI_2_, the effect on PbI_2_ was observed by atomic force microscopy (AFM) as the substitute. Figure 4 shows an AFM image of the surface of the PbI_2_ film. Comparing the films with/without co-evaporant, the film without co-evaporant showed the dispersion of grain sizes, whereas the film with co-evaporant showed the obviously larger grain sizes than the former. Correspondingly, the surface roughness Sq of the film also increased from 7.30 nm to 27.9 nm. This is the first report for the effect of the co-evaporant induced crystallization of inorganic thin films.

The reason for the improvement of *J*sc and corresponding IPCE in Fig. 3a and b can be partially attributed to the improvement of absorption in Fig. 3c, which suggests that the increase of roughness of SnI_2_ film promoted the reaction with GI. Another reason is suggested by the improvement of internal quantum efficiency (IQE) in Fig. 3d that the increase of the perovskite crystal size resulting from the grain size of SnI_2_ suppressed charge recombination^18–26^. As described above, we demonstrated that the co-evaporant induced crystallization is useful even for crystallization of inorganic thin films and improvement of *J*_sc_ of perovskite solar cells.

## Conclusions

In summary, we have revealed that, in spite of disadvantageous tolerance factor, guanidine hydro-iodide, which does not react with PbI_2_, reacted with SnI_2_ and the product of the reaction absorbed visible light much wider than the reactants do. By using this product, we succeeded in fabricating and operating the solar cells. This result confirms that the tolerance factor is not alone the key factor to form perovskite structure. Moreover, we demonstrated that GI can be thermally evaporated without problems often occurred by MAI and FAI. We succeeded in improving the short circuit current density of the vacuum deposited devices by using co-evaporant induced crystallization method during SnI_2_ film deposition. This is the first report of the effect of this method on inorganic materials.

## Methods

### Materials and substrates

To try to form perovskite, we used commercially available high purity reagents (PbI_2_ 99.99%, GI > 97.00%: Tokyo Chemical Industry, SnI_2_ 99.99%: AlfaAesar,) as received. C_60_ (Frontier Carbon) were purified by conventional train sublimation. MoO_3_ (99.998%, Alfa Aesar), Alq_3_ (Tris(8-quinolinolato)aluminum, >98%, Tokyo Chemical Industry), and LiF (Alfa Aesar) were used as is obtained. PDMS (polydimethylsiloxane, Shin-Etsu Silicones, KF96–50cs) was used as a co-evaporant. Indium tin oxide (ITO, thickness: 150 nm, resistivity: 10 Ω/cm^2^) coated 0.7-mm-thick glass substrates were pre-cleaned and patterned by techno Print.

### Film fabrication from solution

A solution of GSnI_3_ was prepared at a concentration of 1.5 × 10^−5^ mol/ml by dissolving SnI_2_ and GI in acetone at a molar ratio of 1:1. In dry atmosphere (dew point was about −40 °C), the solution was dropped onto quartz glass with a surface area of 4.5 cm^2^ and dried at 25 °C. The solution of 0.5 ml and of 1.5 ml were used for the sample preparation of UV-Vis absorption and of XRD measurement, respectively.

### Device fabrication

ITO coated glass substrates were treated by air-plasma in PIB-20 Ion-bombarder (Vacuum Devices). Vacuum deposition was carried out in a custom-designed vacuum chamber (Katagiri engineering and M&Y systems). After introducing the substrates into the vacuum chamber, entire cell fabrication was completed without exposing to the air. All vacuum instruments were connected with oil-free vacuum pumps. Film thickness and the deposition rate were observed using calibrated quartz crystal microbalances equipped with a computer monitoring system (CRTM-9000G/Depoview, Ulvac).

### Device characterization

Device characterizations were performed in a glove-box connected to the vacuum chamber and completed without exposing the devices to the air after the device fabrication. The glove-box (UL-800A, Unico) was filled with N_2_ gas, equipped with a circulation gas purifier (CM-200, Unico). Under normal conditions, the oxygen and water contents were around 1 ppm or less. *J–V* curves were obtained with a custom-designed probe box (Epitec), a Precision Source/Measure Unit (2912B, Agilent), and solar cell measurement software (W32-B2900SOL4-N, Sunrise) under AM 1.5 G one sun illumination from a solar simulator (HAL-320W, Asahi Spectra). IPCE spectra were also measured with the same system under illumination of monochromatic light from a monochromator (CMS-250, Asahi Spectra) and white light bias from LED light source (SLA-100A, Sigma Koki). The temperature of the cells was kept at 25 °C using a Peltier cool plate (CP-085, Scinics). The actual area of each fabricated device was 1.21 or 0.09 cm^2^, and the active area during the characterization was defined as 1.00 or 0.04 cm^2^, respectively, by a precision aperture mask positioned directly on the substrate side of the cell.

### Film characterizations

UV-vis measurement was performed after exposing the devices to the air with a UV/Vis/IR spectrophotometer (V-670, Jasco) equipped with an integrating sphere unit (ISN-723, Jasco). Atomic force microscopy image was obtained in the air by using Probe Station AFM5000II and AFM Unit AFM5100N (Hitachi High Technologies). XRD pattern was obtained in the air by using SmartLab (Rigaku). Powder diffraction pattern was solved by using Crystal Cracker software (Dr. Kurt Leinenweber).
